# Welfare Genome Project: A Participatory Korean Personal Genome Project With Free Health Check-Up and Genetic Report Followed by Counseling

**DOI:** 10.3389/fgene.2021.633731

**Published:** 2021-02-09

**Authors:** Yeonsu Jeon, Sungwon Jeon, Asta Blazyte, Yeo Jin Kim, Jasmin Junseo Lee, Youngjune Bhak, Yun Sung Cho, Yeshin Park, Eui-Kyu Noh, Andrea Manica, Jeremy S. Edwards, Dan Bolser, Sukyeon Kim, Yuji Lee, Changhan Yoon, Semin Lee, Byung Chul Kim, Neung Hwa Park, Jong Bhak

**Affiliations:** ^1^Korean Genomics Center (KOGIC), Ulsan National Institute of Science and Technology (UNIST), Ulsan, South Korea; ^2^Department of Biomedical Engineering, College of Information-Bio Convergence Engineering, Ulsan National Institute of Science and Technology (UNIST), Ulsan, South Korea; ^3^Clinomics Inc., Ulsan, South Korea; ^4^Human Biology Program, Faculty of Arts and Sciences, University of Toronto, Toronto, ON, Canada; ^5^Department of Medical Sciences, Graduate School of Ajou University School, Suwon, South Korea; ^6^Department of Hematology and Oncology, Ulsan University Hospital, University of Ulsan College of Medicine, Ulsan, South Korea; ^7^Department of Zoology, University of Cambridge, Cambridge, United Kingdom; ^8^Department of Chemistry and Chemical Biology, University of New Mexico Comprehensive Cancer Center, University of New Mexico, Albuquerque, NM, United States; ^9^Geromics Ltd., Cambridge, United Kingdom; ^10^Department of Internal Medicine, Ulsan University Hospital, University of Ulsan College of Medicine, Ulsan, South Korea; ^11^Personal Genomics Institute (PGI), Genome Research Foundation (GRF), Osong, South Korea

**Keywords:** genomics, personal genome project, Korean genome project, population study, integrated healthcare, genetic report

## Abstract

The Welfare Genome Project (WGP) provided 1,000 healthy Korean volunteers with detailed genetic and health reports to test the social perception of integrating personal genetic and healthcare data at a large-scale. WGP was launched in 2016 in the Ulsan Metropolitan City as the first large-scale genome project with public participation in Korea. The project produced a set of genetic materials, genotype information, clinical data, and lifestyle survey answers from participants aged 20–96. As compensation, the participants received a free general health check-up on 110 clinical traits, accompanied by a genetic report of their genotypes followed by genetic counseling. In a follow-up survey, 91.0% of the participants indicated that their genetic reports motivated them to improve their health. Overall, WGP expanded not only the general awareness of genomics, DNA sequencing technologies, bioinformatics, and bioethics regulations among all the parties involved, but also the general public’s understanding of how genome projects can indirectly benefit their health and lifestyle management. WGP established a data construction framework for not only scientific research but also the welfare of participants. In the future, the WGP framework can help lay the groundwork for a new personalized healthcare system that is seamlessly integrated with existing public medical infrastructure.

## Introduction

Next-generation sequencing technologies have promoted a variety of personal genome projects (Ball et al., 2012, 2014; Oleksyk et al., 2015; Walter et al., 2015; Cho et al., 2016) aiming to map and analyze whole genome information concerning personal characteristics, population structure, disease risk, and even ancestry prediction (Supplementary Table 1). To this date there are more than three dozens of national-ethnic genome projects worldwide, sequencing human genomes or exomes with diverse goals in various stages of implementation; a number of countries such as the United Kingdom, China, and South Korea have carried out multiple such initiatives (Supplementary Table 1). The Personal Genome Project (PGP) from Harvard Medical School led by George Church (Church, 2005) laid the foundation for all of these independent projects by promoting a voluntary individual driven data collection and sharing by the general public. In a way, it reflects the universal trend of democratizing many aspects of human society and tries to provide personal health benefits based on each person’s sovereignty over his or her own genomic and clinical data. Currently, however, most genome projects are top-down and driven by government initiatives. The first such government-led project to address genomic diversity on a population scale was UK10K launched in 2010 (Walter et al., 2015). It was a British government-funded collaboration with existing projects [TwinsUK (Moayyeri et al., 2013) and ALSPAC (Boyd et al., 2013) cohorts]. UK10K almost entirely focused on diseases such as autism, schizophrenia, and rare conditions. Many subsequent government projects all across the world took similar trajectories: the 100,000 Genomes Project^1^ by Genomics England focused on patients with cancer and rare diseases, Genome Canada^2^ collected 10,000 rare disease trios, and the Ashkenazi Genome Consortium gathered most of their samples from schizophrenia patients (Lencz et al., 2018). More recently, the United Kingdom’s 100,000 Genomes Project approach was adapted by the Taiwanese G2020 Population Genomics Pilot project^3^ aiming at 10,000 participants, while in China several complex disease cohorts were combined for a large-scale ChinaMAP project (Cao et al., 2020).

In addition to disease-related large-scale genome projects, population structure studies have also been initiated as a popular tool to understand the history of human migration and gene flow. Many projects have emerged worldwide for this purpose, acquiring their samples through general population recruitment (Gudbjartsson et al., 2015; Leitsalu et al., 2015; Nagasaki et al., 2015; Ameur et al., 2017; Jeon et al., 2020; Supplementary Table 1). The practical goal and outcome of such non-disease genome projects are to collect and bank as much genomic data as possible for subsequent uses, such as the study of fine stratification of populations. While all these public projects are designed to eventually help the participants and researchers, they are often, as noted by the Precision Medicine Initiative Working Group in 2015 (Hudson et al., 2015), unidirectional in terms of benefits and data sharing from the participants’ point of view. Recently, however, large-scale projects such as “All of Us”^4^ and the “100,000 Genomes Project” (see text footnote 1) released the data generated during the study back to the participants or offered the participants some kind of welfare benefit, including disease diagnosis (Supplementary Table 1). Here, we present the Welfare Genome Project (WGP) funded by the Korean government and the cities of Ulsan and Miryang. It was launched in 2016 in two adjacent cities, Ulsan and Miryang. From the start, WGP had the purpose of providing benefits to the participants. Since all participants are fairly homogeneous in terms of ethnicity and free from serious diseases, their genomic and clinical data can be used as the first standard dataset for a healthy Korean population (∼51 million people^5^).

Ulsan is a metropolitan city in the southeast of Korea with a population of 1.1 million^6^. Excluding Seoul, the capital, six major cities hold a special “metropolitan” status based on comprehensive consideration of their population size, land area, location, and socioeconomic status (Korea Mois, 2020). The industrialization of Korea attracted people from all over the nation to work in numerous factories and global corporations located in Ulsan, such as the Hyundai group. Adjacent to Ulsan is Miryang, a landlocked agricultural city with a population of approximately 100,000 (see text footnote 6). In Korea, conducting genomic sequencing commercially or privately to provide genetic reports is restricted under the regulation of the Bioethics and Safety Act. However, providing such reports to the scientific research participants is allowed if the participants express their interest in receiving the genetic reports. Therefore, WGP is also a social experiment acquiring preliminary insight on whether providing genetic reports can be beneficial to the general public in a particular legal and cultural system. WGP aims to disseminate technical information such as why a welfare-oriented genome project was initiated, what procedures were developed under Korea’s healthcare environment, and how participants were rewarded for their participation.

## Welfare Genome Project Structure

Welfare Genome Project is the first research-based participatory personal genome project in Korea. The project collected and banked genomic, clinical, and lifestyle data from 1,000 healthy volunteers and provided them with a combined genetic and health information service. The genome project was designed to find ways to maximize health and welfare benefits to citizens of participating cities. It was designed, managed, and carried out by the Korean Genomics Center (KOGIC) at the Ulsan National Institute of Science and Technology (UNIST) and the Ulsan University Hospital (UUH) supported by the cities of Ulsan and Miryang. Health check-ups, sample collection, and sample banking were handled by UUH. The generation of personal genetic reports (PGR), screening for common disease risks, physical characteristics, drug responses, and rare disease-associated variants, were carried out by external genomics companies (Supplementary Tables 2–5). WGP was not a cohort study and the participants were not asked to consent to be contacted again for further research at the time of enrollment. Even though all participants were healthy at the time of enrollment, they received an extensive free health check-up at UUH. WGP’s recruitment was carried out by mass media advertisements such as TV commercials and banners in public gathering places, with support from local governments in Ulsan and Miryang. The overall response from the media, cities, and citizens was quite favorable.

## Welfare Genome Project Procedure

**FIGURE 1:**
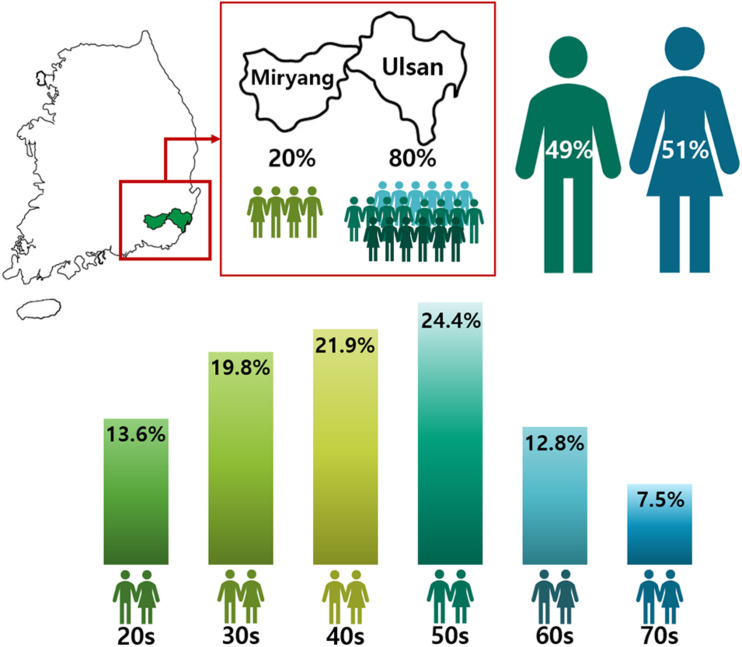
Gender and Age distribution in the Welfare Genome Project. The map shows regions sampled in South Korea. 80% of the participants were from Ulsan, while the remaining 20% were from Miryang. The numbers in the figure show the distribution by gender and age in the Welfare Genome Project.

**FIGURE 2:**
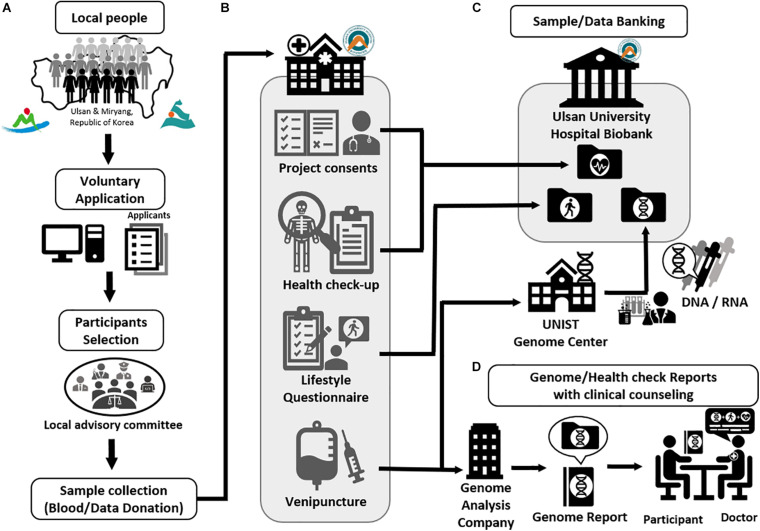
The procedure of Welfare Genome Project. **(A)** Recruitment of participants. **(B)** Sample collection (Blood/Data donation) **(C)** Sample and data banking to Ulsan University Hospital Biobank. **(D)** Providing genetic and health check reports with clinical counseling.

The application procedure was carried out online^7^. Since there was a capacity of 1,000 participants, volunteers from Ulsan and Miryang were selected by a competitive rate of 4.9:1. After classification into respective gender and age groups, participants were randomly selected from their groups to allocate and distribute them as evenly as possible (Figure 1). All participants signed an informed consent form and agreed to; (i) provide blood-derived materials such as DNA and RNA, (ii) make clinical information derived from their health check-up available, and (iii) answer a lifestyle questionnaire (Figure 2). The information provided was banked in the Ulsan University Hospital Biobank. The participants then underwent an extensive health check-up covering 110 clinical traits (Supplementary Table 6), answered a lifestyle-based questionnaire with 137 questions (Supplementary Table 7), and donated 35 ml of blood. The entire process was completed in one visit to UUH (Figure 2).

Approximately 30 days after the initial visit to UUH, the participants were informed of the completion of their PGR, and the participants were required to schedule the final hospital visit for the dissemination of PGR and health check-up results. Doctors provided the participants with their genotype information and explained disease risk scores that are relevant to healthcare and lifestyle, ensuring that the participants received information from medical experts, avoiding possible misinterpretation of the results. On average, it took 45 days from the blood donation to the final consultation, depending on the participant’s availability.

The blood-derived materials, clinical information, lifestyle questionnaire answers, and data generated from them, were transferred to the UUH Biobank. The blood-derived materials were separated into derivatives such as plasma and serum, then anonymized by barcodes using an anonymization program provided by the Korea Centers for Disease Control and Prevention (KCDC) to prevent personal identification by researchers. The blood-derived materials were banked after being stored in a liquid nitrogen tank and deep freezer in the UUH Biobank. The participants’ genotype data were banked together with DNA and RNA at the UUH Biobank.

## Benefits for Participants: Personal Genetic Report

The number of traits examined through PGR for the participants differed by year (Supplementary Table 2). The one in 2016 covered 46 common disease risks, five physical characteristics, five drug responses, and 44 rare diseases (Supplementary Figure 1 and Supplementary Tables 2, 3). The PGR in 2017 contained 50 common disease risks, eleven drug responses, 27 personal characteristics (such as vitamin and caffeine metabolism), and 24 hereditary diseases (Supplementary Tables 2, 4). In 2018, it contained 38 common disease risks, five physical characteristics, five drug responses, and 44 rare diseases (Supplementary Tables 2, 5).

The prevalence of the disease, as well as the participant’s relative risk, was also provided for reference. The participant’s relative risks in the report were subdivided into three to four different risk levels. The risk levels were defined by pathogenicity of genotypes. In 2016 and 2018 there were four levels (management required, elevated, moderate, or mild) (Supplementary Figure 1), but in 2017 the report was simplified to have three risk levels (intensive management required, attention required, and general management). However, critical traits, including the responses to drugs such as warfarin or anticancer drugs and predisposition to myocardial infarction, obesity, arthritis (osteoarthritis and rheumatoid arthritis), atopic dermatitis, alcohol dependence and type 2 diabetes as well as myopia-risk, were consistently covered by all the PGR versions. Furthermore, we screened for and provided the participants with the genotypes of certain disease-related genes, such as *APOB* for familial hypercholesterolemia type B and *AGL* for glycogen storage type III, *CFTR* for cystic fibrosis, and *VWF* for Von Willebrand disease each year.

## Benefits for Participants: Health Check-Up and Report

The participants received an extensive free health check-up. A health report was provided to the participants, which evaluated 110 clinical traits (79 of which were quantitative) and included results from eleven physical traits (i.e., obesity and blood pressure), as well as hematological quantitative results from 16 categories (i.e., blood sugar and thyroid hormones levels). In comparison, the National Health Insurance Corporation (NHIS) health check-up of Korea screens for 17 categories^8^, several of which are fundamental to one’s health status and are also present in the WGP health check-up. However, while WGP assessed every patient for all of the categories regardless of their physical characteristics, NHIS has eight items that are sex and age-specific. For instance, WGP tested every patient for hepatitis B, while NHIS only tests those who are 40 and younger as they are considered to be at a higher risk.

## Participants’ Response to the Survey

After the completion of the project, participants were surveyed on their participation experience (Supplementary Figure 2). Most participants (96.1%) recognized the necessity of WGP (Supplementary Figure 2A) and 87.9% were aware of how genetics affects disease outcomes (Supplementary Figure 2B). Around 92.6% of the participants were satisfied with checking their health (Supplementary Figure 2C) and nearly the same number of participants (92.5%) thought their health information was delivered well (47.8%) or very well (44.7%) (Supplementary Figure 2D). Unsurprisingly, participants were interested to apply genetic information to understand their health since 63.1% of the respondents found PGR to be the most satisfying part of the participation (Supplementary Figure 2E). Moreover, the majority of participants were positive or enthusiastic about donating their blood samples (93.3%) (Supplementary Figure 2F) and clinical data (92.6%) (Supplementary Figure 2G). Approximately 94.7% of the participants answered that knowing their genetic information can positively impact their life (Supplementary Figure 2H), and 91.0% responded that this project encouraged them to consider making lifestyle changes toward health improvement (Supplementary Figure 2I). Overall, the majority of the Ulsan and Miryang citizens were satisfied with the participation experience and services provided.

## Extension of the Welfare Genome Project

The samples banked to the Biobank of Ulsan University Hospital already proved to be a valuable resource to the scientific community, as they have been used in Korea1K, the biggest South Korean population genome project. Jeon et al. (2020) sequenced the whole genome of 696 out of 1,000 samples banked by WGP and used the 79 quantitative clinical traits to investigate genome-wide associations between the variants and the clinical information. Another application demonstrated by the study was the development of a Korean panel of normals for cancer genomics researches, which is an invaluable resource when a matched healthy control from the same individual is not available (e.g., leukemia).

Furthermore, one of the most impactful applications of such a large-scale WGS is building a genotype imputation panel. Since an imputation panel can be used to impute unknown genotypes, this can be a useful resource for many genome-wide association studies and clinical studies. The Korea1K genotype imputation panel, which includes a large number of samples from the same population (Koreans), also showed a strong increase in predictive power (Jeon et al., 2020).

Due to a relatively even age stratification among the samples and both DNA and RNA availability, WGP samples can also be used for aging research *via* gene expression and methylation profiling. Other potential applications can utilize the banked clinical information and lifestyle survey results focusing on genotype effect on simple (e.g., lactose intolerance) and complex phenotypes (e.g., obesity, susceptibility to cancers). The genomic data generated is a useful resource for interpopulation comparisons. Additionally, the large number of genomes can give enough resolution for population structure estimation. The publication by Jeon et al. (2020) is just a first example of WGP samples being successfully used for future research.

## Discussion

Despite the support and positive outlook from the general public, WGP encountered two major limitations. The first shortcoming was the lack of genetic counselors who could perform a consultation at the recruiting stage and later interpret and explain the genetic report results to the participants at the report stage. While general practitioners can partially fill in the counseling role, their genetic interpretations may lack depth. This becomes even of higher importance when we consider the recent estimates (2006–2020), where the general population recruited to omics projects in most studies (64%) does not have a sufficient level of knowledge about genomics (Calabrò et al., 2020). However, the competence of general practitioners involved in genome projects has already been demonstrated by Estonian Biobank, another project recruiting the general population (Leitsalu et al., 2015). Secondly, genomic data are classified as personal information, which South Korea strictly regulates *via* the Bioethics and Safety Act^9^. Initially, the Act emphasizes the need for consent form the individual as a human subject of research. Upon its donation to a human material biobank, genetic data must be anonymized to prevent personal identification (Article 44-2) (see text footnote 9). In the case that a subject’s information is breached, the Act states that one cannot be discriminated against based on their genetic information (Article 46-1) (see text footnote 9). Although such laws serve to ensure ethical use and protect the security of participants’ genetic data, the ambiguity in their interpretation creates a non-sensical situation where subjects may participate in WGP-like projects and receive the genetic report but are prohibited from receiving their genome sequence. As in FinnGen (Ritari et al., 2020)^10^ and All of United States (see text footnote 4) projects, the WGP genomic data were planned to be integrated into healthcare systems (Supplementary Table 1), but it was impossible by the time of WGP completion. Inspired by this limitation, in 2020, after the project was completed, Ulsan city government was motivated to establish a special Genome Regulation Free Zone status from the government (Ulsan Genome Service Industry Regulation-Free Special Zone), authorizing the transfer of genome information to third parties such as public biobanks and biotech companies as well as returning it to the participants (see text footnote 10). While an exception was made for Ulsan city, current regulations in Korea are still not favorable for the development of personalized medicine, capping the scale and usefulness of projects like WGP.

We showed that both researchers and volunteers can benefit simultaneously by raising the public interest in WGP, especially for the participants who received the health check-up and genetic report, the key factor differentiating WGP from other genome projects. Ultimately, the omics data set of relatively healthy people is important and can be used as universal demographic control data for cancer, rare disease, aging, and population structure studies. As it is important to monitor the public interest and provide continuous education and information on genetic predisposition, we conducted a survey after the project. The responses to the survey suggest that allowing participation in genomic research and providing detailed information about their own genome sequences is an effective way to educate people about genomics and genetic association with diseases. As an outcome of the project, we identified some participants becoming more motivated in taking care of their health than before WGP participation (Supplementary Figure 2I). Perhaps the most important social impact we have discovered was that majority of the participants were quite positive (>90% positive response) about donating their genomic as well as clinical information, and understood how their genome could be used as a resource for healthcare research. The response was consistent with the survey results by the Korea Centers for Disease Control and Prevention (KCDC) (Kim et al., 2020) and comparable to the results of the 100,000 Genome Project^11^ and Qatar Genome Program (QGP) (Qoronfleh et al., 2020). Certainly, it is important to note that our participants’ response was intrinsically biased by the free benefits they received. However, WGP’s survey was only on the participants, while QGP (Qoronfleh et al., 2020) surveyed the general Qatari population with which 71% responded positively toward the genome project. Therefore, the overwhelmingly positive response in our study may be biased but not unprecedented. On the other hand, the scope of the United Kingdom’s 100,000 Genome Project complicates its comparison with WGP. Despite the differences, 73% of the 100,000 Genomes Project participants were positive about their involvement, which is highly consistent with our survey results, and 86% would return to future genomic initiatives (see text footnote 12). Furthermore, one additional aspect of WGP was that companies participated in producing genetic reports by acquiring single nucleotide variant (SNV) information. Although it was limited in terms of traits and the number of SNVs, it was the first case in Korea where commercial entities provided their genetic report products to the general public as a part of this research project.

Additionally, regarding the clear rules for the ethnical use of data and data ownership, participants were clearly informed and agreed to the donation of human materials after accepting the following guideline established by UUH Biobank: “Participants don’t have rights for product development and patent applications based on research results using human materials provided by participants. The research results can be published in academic conferences and journals in the name of the research and participant’s personal information will not be revealed.” However, their close relatives may raise legal issues on the genetic information that will be publicly shared in the future although participants have fully agreed to donate their genomic data for scientific research. This issue may escalate further when a great number of large-scale genome projects are launched in the future. The establishment of unified international standards on consenting and data sharing would partly resolve the legality regarding the personal and family privacy issue of genome projects. However, this is an issue beyond the scope of WGP. Furthermore, as more commercial DNA tests become available, internal security measures cannot completely prevent possible unwanted identification through genome information, since individuals are traceable by genetic similarity. Thus, one should be mindful when acquiring their genetic information.

The representativeness of samples must also be considered carefully. WGP sampling was conducted only in two cities, thus insufficient in representing the genomic diversity of the entire Korean population. While it is a geographically limited approach, the industrial and metropolitan aspects of Ulsan suggest that it attracted people from all over the country, contributing to its genomic diversity. Furthermore, most population genetics studies on Koreans so far indicated that Koreans are genetically homogeneous (Kim et al., 2010; Hong et al., 2014; Cho et al., 2016; Jeon et al., 2020). Thus, we concluded that the possible sampling bias of WGP is not critical. The outcome of this project demonstrates that Korean citizens are fairly open to participation in large-scale personal and public genome projects. If expanded, the WGP model can be applied not only in other Korean provinces, but in other regions worldwide.

Overall, the genomic and clinical data generated by WGP can be a very useful future resource. It can serve as a standardized control set when investigating many diseases. Furthermore, providing a pilot path for genome projects by reducing uncertainty in bioethics regulations could potentially advance the current Korean healthcare system, improving personalized medicine using genetic predispositions and metabolic traits. This may ultimately facilitate society to build a personal healthcare system that is seamlessly integrated with existing public medical infrastructure. For example, a set of genomic and clinical information resources would allow integrating innovative approaches such as polygenic risk score calculation and prediction, which has been shown to improve risk prediction accuracy for patients of coronary artery diseases (Elliott et al., 2020). Therefore, participants may benefit from WGP over their lifetime by acquiring their genetic information especially when it is integrated into a preventive healthcare service in the future.

## Data Availability Statement

The datasets presented in this article are not readily available because the datasets (human derived materials) generated and analyzed during the current study are available in the repository, https://www.uuh.ulsan.kr/kr/index.php?pCode=bio_data, by requesting the Biobank of Ulsan University Hospital. Requests to access the datasets should be directed to https://www.uuh.ulsan.kr/kr/index.php?pCode=bio_data.

## Ethics Statement

The project was approved by the Ulsan National Institute of Science and Technology Institutional Review Board (UNISTIRB-16-13-C). All of the participants signed informed consents which included a statement on their de-identified data being potentially published.

## Author Contributions

YC, YP, SL, and JB designed the business. E-KN and NP managed the samples and business. YJ, SJ, AB, YK, JL, and JB wrote the manuscript. YJ, YK, AB, SK, YL, and CY visualized the results. YB, AM, JE, DB, BK, SL, and JB provided feedback and editorial support on the manuscript. All authors contributed to the article and approved the submitted version.

## Conflict of Interest

YK, YC, and YP were employed by the company Clinomics Inc. DB was employed by the company Geromics Ltd. BK and JB are the CEOs of the company Clinomics Inc. The remaining authors declare that the research was conducted in the absence of any commercial or financial relationships that could be construed as a potential conflict of interest.

## Footnotes

1 https://www.genomicsengland.co.uk/the-journey-to-100000-genomes

2 https://www.genomecanada.ca/en/programs/precision-health-strategy-canada-think-big-start-small-learn-fast

3 https://enews.nhri.org.tw/en/?p=858

4 https://allofus.nih.gov/about/all-us-research-program-overview

5 https://data.worldbank.org/indicator/SP.POP.TOTL

6 https://www.citypopulation.de/en/southkorea/cities

7 http://ulsan1000.genomekorea.kr/

8 https://hi.nhis.or.kr/aa/ggpaa001/ggpaa001_m01.do

9 https://elaw.klri.re.kr/eng_mobile/viewer.do?hseq=33442&type=part&key=36

10 https://www.finngen.fi/en/goals_and_benefits

11 https://www.geneticalliance.org.uk/wp-content/uploads/2019/07/Patient-Experience-100000GP.pdf
