# Supplementary material for: Welfare Genome Project: A Participatory Korean Personal Genome Project With Free Health Check-Up and Genetic Report Followed by Counseling
Source: Front Genet. 2021 Feb 9;12:633731. doi: 10.3389/fgene.2021.633731 (PMC7900555; doi:10.3389/fgene.2021.633731)
Supplement: Supplementary file 1 [file Data_Sheet_1.DOCX]

Supplementary Material


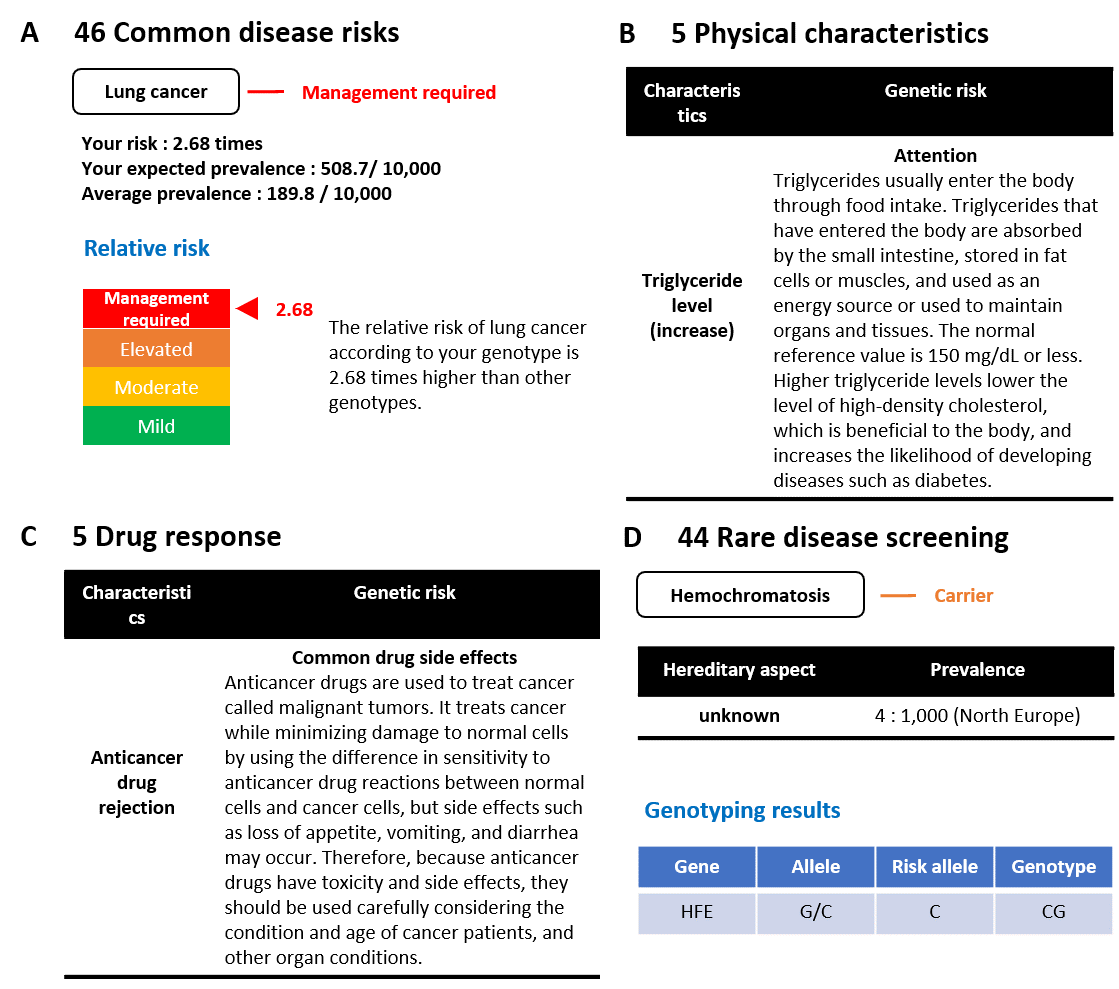


**Supplementary Figure S1. Sample of genetic report received by participants.**

The figures show a sample of the genetic report which consists of four major sections: (A) common disease risks, (B) physical characteristics, (C) drug response, and (D) rare disease screening.

**
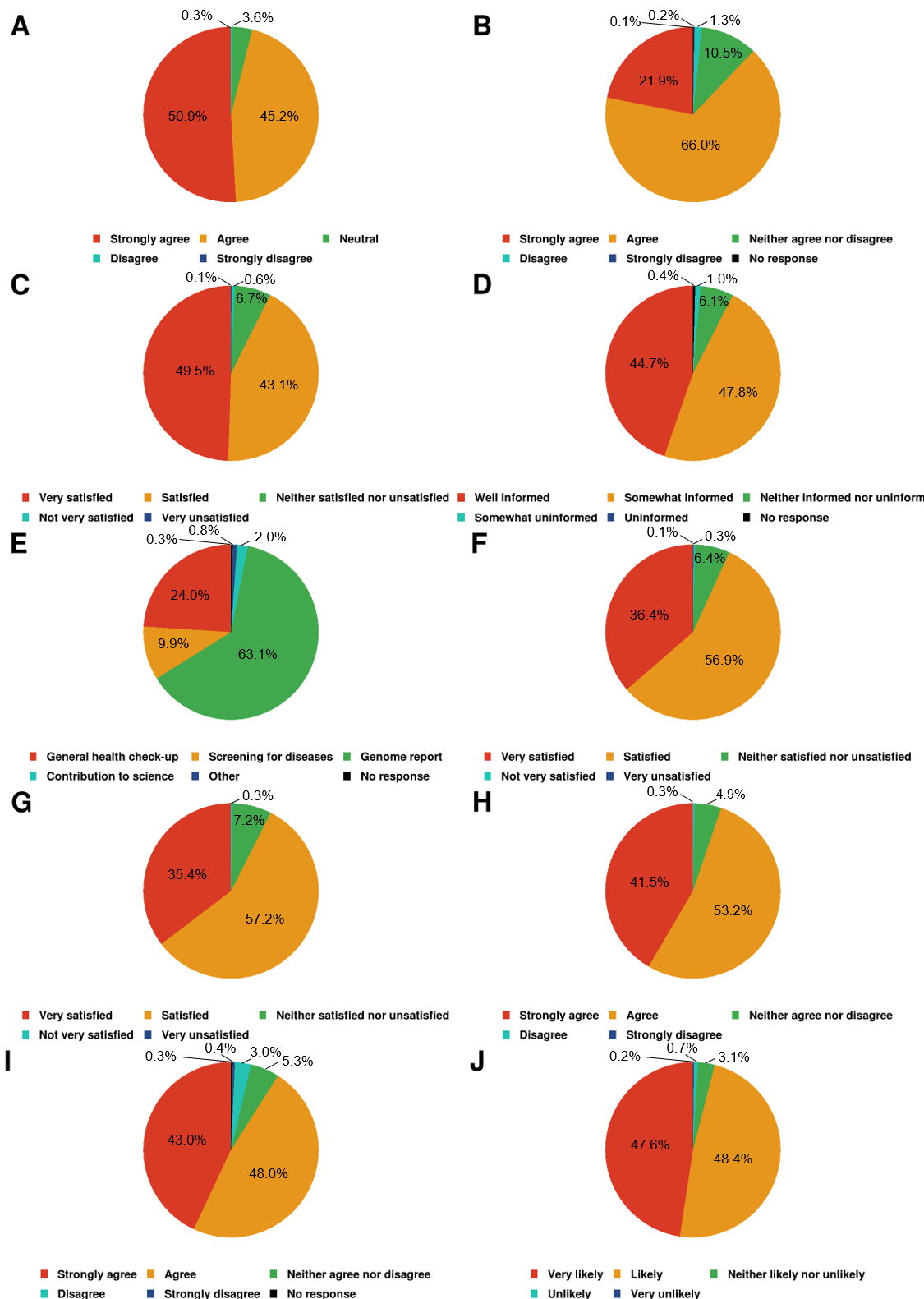
 Supplementary Figure S2. Survey results on participation experience**

Pie charts (A-J) show the proportion of survey answers from participants. Colors indicate the participants’ answers. The questions in the survey are (A) I believe that the Welfare Genome Project for the citizens of Ulsan and Miryang through genomics is necessary (B) I believe that genetics affects disease outcomes (C) How satisfied were you with checking your health via your genetic information? (D) How well were you informed on your current state of health? (E) What was the most satisfying part about participating in the Welfare Genome Project? (F) How would you rate your experience donating your blood/genome for science? (G) How was your experience with venipuncture for the health check-up and sample donation? (H) I believe that genomics will bring positive influence to humankind through technology such as biotechnology and the pharmaceutical industry (I) The results motivate me to take steps towards improving my health (J) How likely is it for you to recommend participation in the Welfare Genome Project to others?
